# Modular organization in the reductive evolution of protein-protein interaction networks

**DOI:** 10.1186/gb-2007-8-5-r94

**Published:** 2007-05-28

**Authors:** Javier Tamames, Andrés Moya, Alfonso Valencia

**Affiliations:** 1 Instituto Cavanilles de Biodiversidad y Biología Evolutiva, Universitat de València, 46071 Valencia, Spain; 2 Structural and Computational Biology Programme, Spanish National Cancer Research Centre (CNIO), 28029 Madrid, Spain

## Abstract

**Background:**

The variation in the sizes of the genomes of distinct life forms remains somewhat puzzling. The organization of proteins into domains and the different mechanisms that regulate gene expression are two factors that potentially increase the capacity of genomes to create more complex systems. High-throughput protein interaction data now make it possible to examine the additional complexity generated by the way that protein interactions are organized.

**Results:**

We have studied the reduction in genome size of *Buchnera *compared to its close relative *Escherichia coli*. In this well defined evolutionary scenario, we found that among all the properties of the protein interaction networks, it is the organization of networks into modules that seems to be directly related to the evolutionary process of genome reduction.

**Conclusion:**

In *Buchnera*, the apparently non-random reduction of the modular structure of the networks and the retention of essential characteristics of the interaction network indicate that the roles of proteins within the interaction network are important in the reductive process.

## Abstract 2

Analysis of the reduction in genome size of *Buchnera aphidicola *from its common ancestor *E. coli *shows that the organization of networks into modules is the property that seems to be directly related with the evolutionary process of genome reduction.

## Background

Bacterial endosymbionts of insects, such as *Buchnera aphidicola *[1,2], *Blochmannia floridanus *[3] and *Wigglesworthia glossinidia *[4], are paradigms of reductive evolution. These bacteria live in a stable and isolated environment, the bacteriocyte of insects, where the host provides most of their nutritional requirements. As a consequence, the genomes of these bacteria have undergone a process of reduction, losing around 90% of their ancestral genes. These endosymbionts also fail to acquire new genes due to their incapacity to incorporate DNA via lateral gene transfer and their isolated environment. Nevertheless, although their genomes represent a subset of the genome of their ancestors, these gamma-proteobacteria remain closely related to *Escherichia coli *(98% of the genes in *Buchnera *have clear orthologues in *E. coli*). Accordingly, the process of genome shrinkage that these species have undergone has been well documented in terms of the evolution of the corresponding protein families [1,2].

Recent research indicates that the capacity of an organism for adaptation depends not only on the properties of its individual molecular components, but also on the structure and organization of its underlying network of molecular interactions. Indeed, it was recently proposed that the modular organization of the network of interactions is necessary to adapt to changing environments [5]. In such a modular system, the compartmentalization of a set of interactions that are both closely interconnected and remain weakly connected to other components in the artificial environment increases. Accordingly, the organization into so-called modules is favored by constant changes in environmental conditions, highlighting the direct causal relationship between such changes and the increase in network modularity. Nevertheless, this proposal awaits a direct assessment in a real biological system.

Studies on the organization and properties of protein networks have flourished recently thanks to data from high-throughput experiments, for example, two-hybrid screens, pull-down experiments and ChIP-on-chip studies [6-10]. Despite limitations in terms of the extent and quality of the datasets, the results produced have been fundamental in enabling the first studies of network structure to be carried out [7,11]. Such studies have involved the comparison of networks from different origins [12] and the construction of the first models of network behavior and evolution [13,14].

Taking advantage of the two recently published high-throughput protein interaction maps of *E. coli *[9,15], we have performed a study in which we focused on the reductive evolution of the *Buchnera *genome. The comparison between the *E. coli *and *Buchnera *interaction networks was based on the assumed low rate of protein interaction turnover [16] and the weak probability that new interactions would be generated in the restricted conditions in which *Buchnera *lives. Accordingly, it can be assumed that when proteins are conserved between *E. coli *and *Buchnera*, the protein interactions are also likely to be maintained [17]. Therefore, the direct relationship between the genomes, the clear conservation of proteins and the probable similarity of their interactions provides a perfect scenario to assess the consequences of adaptation to a stable and nutrient-rich environment.

*E. coli *is a free-living bacteria known to be capable of adapting to very different environments [18-20]. In contrast, *Buchnera *is an endosymbiotic bacteria living in a very stable medium. As a result, we would expect the *E. coli *network to be more modular than that of *Buchnera*. Hence, reductive evolution might be responsible not only for decreasing the gene repertoire of *Buchnera*, but also for reducing its network modularity. This hypothesis can be tested by comparing the organization of the protein-protein interaction networks of these two species.

## Results and discussion

### Modular structure of the *E. coli *network

Modules are set of components (proteins) with a clear imbalance in favor of internal versus external connections. Therefore, the modularity of a network can be quantified by comparing the number of connections within and between modules. Consequently, the main problem when defining modules is the search for the optimal division of the network that maximizes the ratio between intra- and inter-module connectivities. Several algorithms have been proposed to carry out the task of decomposing networks into their modular components [21-24]. We have used two recently proposed algorithms [23,24] that have been shown to produce optimal decomposition of biological networks. Since both algorithms are based on different approaches, and two different maps of protein-protein interactions of *E. coli *are available [9,15], the validity of the conclusions is relatively independent of the method and the data source. It is important to realize that the values of the modularity coefficients have to be normalized/corrected with respect to the modularity expected in equivalent random networks of the same connectivity, thereby eliminating the effect that the pattern of connections in the network could have on the calculation of its modularity (see Materials and methods).

The results of analyzing the structure of the *E. coli *network show that it is most modular at any level, irrespective of the clustering methods used (see Table S3 in Additional data file 1 for descriptions and results obtained using other clustering approaches for determining modularity). The optimal decompositions render between 10 and 15 modules (Table 1), most of them significant from a functional point of view (see Materials and methods). Some of the modules are quite homogeneous and contain easily discernible functions, that is, protein synthesis (including ribosomal proteins), transcription (RNA polymerase), cell division, DNA synthesis (DNA polymerase), or DNA maintenance, corresponding well to the empirical analysis of the original dataset established by Butland *et al*. [9]. These modules account for more than half of the modularity in the network (Table S1 in Additional data file 1). Other modules contribute less to the global modularity and are composed of proteins with more diverse functions. The overall structure of the network indicates the existence of a central core that is clearly organized into modules of protein interactions, while many other functions or activities associated with this core display less modular structure.

**Table 1:** Values of modularity for *E. coli *and *Buchnera *networks

Dataset	Modules and validation	Q_real_	Q_rand_	Q_norm _(Q_real _- Q_rand_)
**Newman algorithm**				
*E. coli*, Butland dataset	12 (5/10)	0.346	0.244	0.102
*Buchnera*, Butland dataset	7 (3/7)	0.259	0.232	0.027
*Buchnera *constrained, Butland dataset	7 (2/6)	0.182	0.168	0.014
*E. coli*, Arifuzzaman dataset	15 (8/13)	0.409	0.329	0.080
*Buchnera*, Arifuzzaman dataset	10 (4/9)	0.460	0.423	0.037
*Buchnera *constrained, Arifuzzaman dataset	12 (4/10)	0.274	0.265	0.009
*E. coli*, STRING	33 (32/32)	0.670	0.209	0.461
*Buchnera*, STRING	12 (11/11)	0.581	0.272	0.309
*Buchnera *constrained, STRING	14 (11/11)	0.493	0.210	0.283
**Guimerá algorithm**				
*E. coli*, Butland dataset	10 (7/10)	0.357	0.248	0.109
*Buchnera*, Butland dataset	6 (3/5)	0.263	0.237	0.026
*Buchnera *constrained, Butland dataset	8 (2/7)	0.192	0.179	0.013
*E. coli*, Arifuzzaman dataset	12 (6/11)	0.413	0.332	0.081
*Buchnera*, Arifuzzaman dataset	8 (4/8)	0.461	0.432	0.029
*Buchnera *constrained, Arifuzzaman dataset	11 (2/8)	0.266	0.242	0.024
*E. coli*, STRING	19 (17/17)	0.669	0.211	0.458
*Buchnera*, STRING	11(10/10)	0.566	0.277	0.289
*Buchnera *constrained, STRING	9 (7/7)	0.489	0.231	0.258

Modularity is calculated using different algorithms as described in the text for the *E. coli *and *Buchnera *networks. The module validation is indicated between parentheses after the number of modules for each network and this provides information on the number of modules that are statistically significant with regards to the STRING data (see text for details). For instance, 5/10 means that five out of ten modules are significant in terms of STRING interactions. The number of modules validated is sometimes different to the total number of modules, since some modules are too small to be statistically assessed. When using STRING-derived networks, all modules can be validated since the same information was used to construct the network. The table also shows the modularity coefficient (Q) for real and randomized networks, and the normalized modularity coefficient, resulting from the subtraction of the modularity coefficients for real and random modules.

The potential *Buchnera *protein interaction network was obtained by maintaining the connections between the orthologous proteins in *E. coli*. The modular decomposition of the resulting network shows that the *Buchnera *network was always significantly less modular than that of *E. coli *(Table 1). The decrease in the modularity coefficient implies that the network obtained for *Buchnera *is much harder to separate into isolated components than that of *E. coli*. Therefore, we concluded that the process of reducing the genome size (reductive evolution) creates a less compartmentalized network with a smaller degree of modularity.

**Figure 1:**
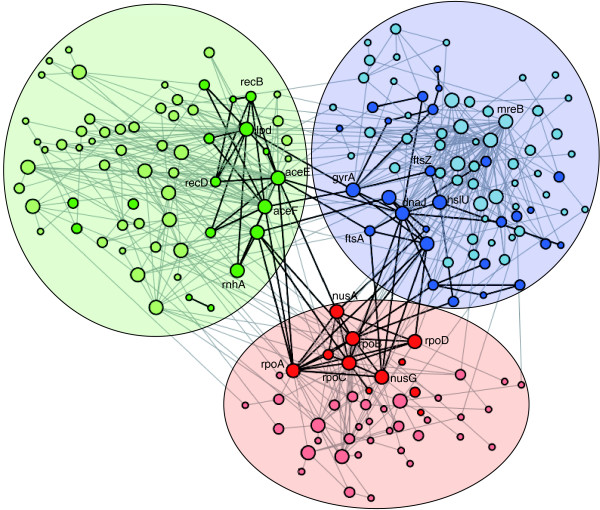
View of three modules of the *E. coli *network. The blue module corresponds to cell division and chaperones. The red module is related to RNA polymerase and the green module involves DNA metabolism. The size of the nodes indicates their absolute degree or number of connections. Conserved nodes in *Buchnera *are shown in darker colors, while conserved connections are shown in thick black lines. Connector hubs are completely conserved, whereas non-hub connectors are deleted in some instances.

An alternative approach is to study the process of module reduction maintaining the modular structure obtained for *E. coli *but deleting the proteins that do not have orthologues in *Buchnera*. In this way, the reduction of the modules originally defined in *E. coli *can be assessed. We found that the ensuing 'constrained' decomposition of the *Buchnera *network is also less modular than that of *E. coli*. Indeed, the modularity observed is similar to that observed when the *Buchnera *network was decomposed independently (Table 1). Furthermore, with the exception of the module containing ribosomal proteins, the modules in the 'constrained' network are significantly smaller than those in E. *coli*. The deletion involves between 70% and 91% of the nodes and, interestingly, the set of conserved nodes often consists of those involved in the connection between modules (Figure 1).

Nevertheless, the coefficients are low in all cases. In *E. coli*, they are around 0.1, indicating little modularity (high modularity is achieved when the coefficient reaches values around 0.3). The coefficients are close to zero in all *Buchnera *networks, indicating that modularity has been almost completely lost in these networks.

### The role of the nodes in the reduction of the modular structure of the network

The connections between modules in the *E. coli *network are dominated by non-hub connectors, that is, nodes with an average number of links within their module but that are well connected to other modules [23]. These nodes account for more than 80% of the connections between modules. The remaining connections are made by connector hubs with strong links both within and between modules but that are, in turn, weakly connected between themselves (examples of connector hubs are peptidyl-prolyl cis/trans isomerase *tig *and pyruvate dehydrogenase *aceE*). This is characteristic of a feature known as dissortativity [11], which has been documented in several other biological networks[21]. There is extensive communication between modules in the *E. coli *network and this is mainly based on the links provided by non-hub connectors.

**Figure 2:**
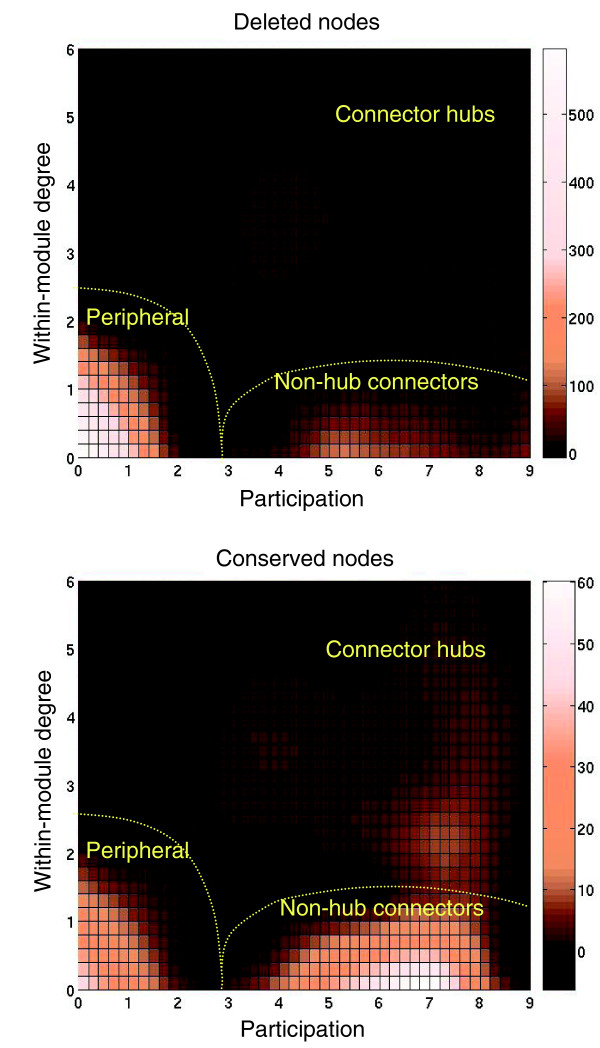
Density map of the role of the nodes in the *E. coli *network that are conserved or deleted in *Buchnera*, according to the procedure described in [23]. The degree of participation measures the connection of a given node with the nodes from modules other than its own. The within-module degree measures the connection of the node with other nodes within its own module. Peripheral nodes show both low participation and low within-module degree. Non-hub connectors participate significantly and with a low degree of within-module connections, while connector hubs have both high participation and high degree of within-module connections [23]. Connector hubs and non-hub connectors are mainly conserved in the *Buchnera *network, while the deletion of nodes mainly affects peripheral nodes. The measures are calculated as in [23], based on the modular division of the *E. coli *network obtained from the Butland dataset. The scale refers to the number of nodes in each position.

In the constrained reduced *Buchnera *network, it is apparent that the number of peripheral nodes has diminished. While there was less than average loss of non-hub connectors, connector hubs were almost completely preserved (Figure 2). Therefore, connector hubs appear to create a highly preserved backbone of interactions. This emphasizes the crucial importance of connector hubs in maintaining the integrity of the protein network, in contrast to the findings from studies of metabolic networks [21].

### The reduction of network modularity and of the overall properties of the network

Reduction of modularity affects certain topological aspects of the network. For simplicity, we restrict our analysis to the results for the Butland dataset, since the results for the Arifuzzaman [15] dataset are very similar. The analysis of connectivity shows that the *E. coli *and *Buchnera *networks follow a power-law distribution with exponents (*γ*) of 2.25 for *E. coli *and 2.03 for *Buchnera*. The smaller exponent in *Buchnera *indicates that hubs are more prevalent in the network, since they are in contact with a larger proportion of nodes. This highlights the relevance of connector hubs, which produce a more compact network in *Buchnera*, as reflected by the average number of links per node (6.07 link per node in *Buchnera *versus 4.16 in *E. coli*) and the smaller diameter of the *Buchnera *network (2.821 versus 3.607 for *E. coli*). Both networks are almost completely connected, which means that there are very few nodes in islands not linked to the main component. In both networks, isolated nodes constitute just 2% of the total number of nodes. Additionally, the length of the paths crossing the network remains unaltered, and only 60 of a possible 37,408 paths were longer in *Buchnera *than in *E. coli*, with a difference of just one node. Therefore, rather than fragmenting the network, the removal of nodes and links in the *Buchnera *network maintains the global topology of the network, preserving the main interaction backbone. The preferential deletion of connections between peripheral nodes that lie outside of the core of the network creates an apparent enrichment of densely connected motifs in *Buchnera*, particularly when the relative proportions are considered (Table S1 in Additional data file 1).

When nodes were randomly removed from the *E. coli *network until it reached a size equivalent to that of *Buchnera*, the organization of the network was completely lost. The resulting network is fragmented into a myriad of small components (islands), each with few isolated nodes. This is an important indication of how node deletion during reductive evolution has been accomplished in a controlled manner that preserves the network organization and the cross-talk between the remaining processes.

## Conclusion

We compare the structure of two independent sets of experimentally derived interactions for *E. coli *with the deduced structure of interactions for the closely related *Buchnera *genome. Thus, the reductive evolution followed by *Buchnera*, whereby more than 90% of the ancestral genes have been lost, is correlated with the loss of modularity of the protein interaction network. Nevertheless, the rest of the characteristics of the network in *Buchnera *essentially remain unchanged. These observations provide an initial model to understand reductive evolution, adaptation to environments and network organization. As in previous analyses of network structure, it is clear that, in this early phase, the models will benefit greatly from additional information from other genomes, and from an overall improvement in the quality of the proteomic experiments. Nevertheless, even bearing these limitations in mind, it is possible to see how the reduced modularity in the *Buchnera *genome is caused by the partial deletion of nodes in regions that are connected to dense clusters of essential functions in the *E. coli *protein interaction network. This is demonstrated by measuring the modularity in the reduced network. In contrast to what would be expected if the preferentially deleted genes were those participating in a non-modular part of the *E. coli *network, the modularity decreased with respect to the *E. coli *network.

**Figure 3:**
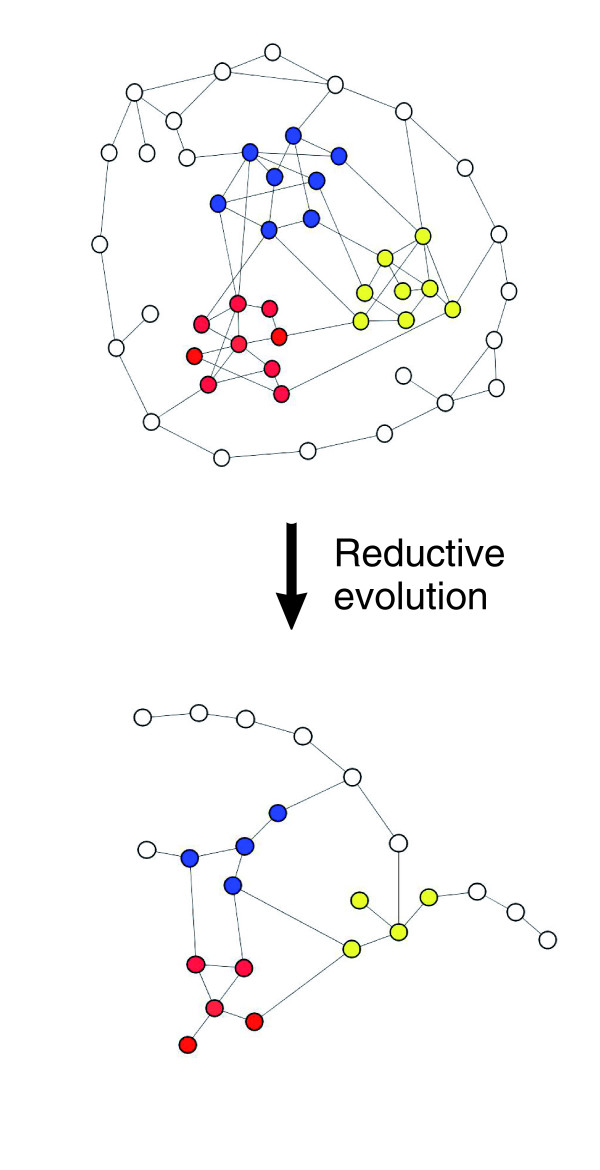
Deletion of interactions may produce reduced modularity. Three modules (red, yellow, blue) are shown, surrounded by a non-modular region. Even if the reduction is higher in peripheral nodes (non-modular region), modularity may decrease since the module structure is lost and only the backbone remains.

The *E. coli *network is apparently composed of a modular core and a mostly non-modular peripheral region. This could imply that, at this level, modular structures are not determinant for the evolution of the network. Reduction of modularity is not achieved by the removal of entire modules (which could even produce an increase in the modularity coefficient), but rather by selective deletion of nodes in the modular parts of the network (Figure 3). In other words, the process of genome reduction apparently involves deleting peripheral regions of the network and the selective loss of proteins forming part of densely packed clusters that are separated into modules. However, it affects the proteins directly implicated in maintaining the connections between modules to a much smaller extent (Figure 2). The result is a very compact network with a smaller diameter, a conserved backbone and an increase in the proportion of densely connected motifs, as well as the preservation of characteristics such as path length and network topology. The way to maintain or increase modularity in reduced networks would be to remove connections between modules and, therefore, communication between processes, which could be highly deleterious. Our conclusion is that the loss of modularity in *Buchnera *networks seems to be mainly related to the conservation of the network backbone, rather than resulting from the loss of adaptability to environmental conditions.

These results might be important in the context of the evolutionary implications of network structure. It has been suggested that the organization of biological networks (interaction and control networks) is a direct product of the simple process of gene duplication and deletion, and that it is not directly subjected to natural selection [16]. The apparently non-random reduction of the modular structure of the networks and the retention of essential characteristics of the interaction network indicate that the roles of proteins within the interaction network are important in the reductive process. Accordingly, the importance of the roles of the proteins must be taken into consideration when discussing the effect of the natural selection on the organization of protein networks.

## Materials and methods

Protein-protein interaction data for *E. coli *were obtained as described in the original studies [9,15].

The first study [9] is based on yeast-based tandem affinity purification (TAP) adapted to *E. coli*. In this procedure, 1,000 *E. coli *open reading frames were tagged (22% of the genome) and their interactions with other proteins within this set were determined. It was possible to determine 5,254 protein-protein interactions, involving 1,264 proteins (Butland dataset). To our knowledge, this was the first set of *E. coli *protein-protein interaction data determined by high-throughput procedures.

The second study [15] was based on producing His-tagged bait proteins; after co-purifying the interacting bait and prey proteins on a Ni^2+^-NTA column, they were identified by mass spectrometry. There were 4,339 *E. coli *proteins tested, for which 11,511 interactions were determined. The authors provided a reliable set of 8,893 of these interactions, involving 2,821 proteins, which were reproducible in the original study (Arifuzzaman dataset). The reliable set was the one used by us in this study.

While both datasets share 983 proteins, only 168 interactions are present in both sources, a situation similar to that observed in yeast [25].

For *E. coli *proteins, orthologues in *B. aphidicola *strain APS (RefSeq NC_002528) were identified by perfoming BLASTP homology searches. To correctly identify orthologues, both proteins must fulfill the following criteria: one is the best hit of the other (best bi-directional hits); the BLASTP E-value must be above 1e-15; and the alignment must span at least 80% of the residues in both proteins. Considering complete genomes, we were able to identify *E. coli *orthologues for 98% of *Buchnera *proteins, while around 90% of *E. coli *protein-coding genes have been deleted from the *Buchnera *genome (*E. coli *strain K-12 contains 4,243 genes; *Buchnera *has 564 genes). For the two sources of data (1,264 *E. coli *proteins in the Butland dataset and 2,821 in the Arifuzzaman dataset), we identified 278 and 260 orthologues in the proteome of *Buchnera*, respectively.

The protein-protein interaction network in *Buchnera *was generated by mapping *E. coli *interactions between conserved proteins in *Buchnera*. The removal of nodes (proteins) implies the removal of all links attached to them. This creates a network of 1,638 interaction pairs for the Butland dataset and 549 for the Arifuzzaman dataset, implying that the latter is enriched in interactions between proteins that are not conserved in *Buchnera*.

We also created a third network based on data from the STRING database [26,27]. STRING contains known and inferred relationships between *E. coli *proteins derived using diverse methods. The version of STRING used in this work involves 3,868 proteins implicated in 33,733 relationships, and it does not include the data from the other two sources. Thus, it comprises an independent set of interactions that can be used to validate the modular decomposition of the networks.

For the networks, the node degree was measured as the number of links for each node. Links were non-directional and corresponded to protein-protein interactions. Protein motifs were identified as described previously [12]. The path length (*l*) between all pairs of nodes was calculated using a standard Dijstra algorithm.

A module is defined as a part of the network with abundant connections between the nodes within it, and less connected to nodes outside the module. The ratio between these two measures (connections within the module and with other modules) defines the modularity coefficient *Q*. The modularity coefficient was calculated as the fraction of edges in the network that connect the nodes in a module minus the expected value of the same quantity in a network, with the same assignment of nodes in modules but with random connections between nodes [5,22,23]:

Q=∑s=1K[lSL−(ds2L)2]
 MathType@MTEF@5@5@+=feaafiart1ev1aaatCvAUfeBSjuyZL2yd9gzLbvyNv2Caerbhv2BYDwAHbqedmvETj2BSbqee0evGueE0jxyaibaiKI8=vI8tuQ8FMI8Gi=hEeeu0xXdbba9frFj0=OqFfea0dXdd9vqai=hGuQ8kuc9pgc9s8qqaq=dirpe0xb9q8qiLsFr0=vr0=vr0dc8meaabaqaciGacaGaaeqabaqadeqadaaakeaacaWGrbGaeyypa0ZaaabCaeaadaWadaqaamaalaaabaGaamiBamaaBaaaleaacaWGtbaabeaaaOqaaiaadYeaaaGaeyOeI0YaaeWaaeaadaWcaaqaaiaadsgadaWgaaWcbaGaam4CaaqabaaakeaacaaIYaGaamitaaaaaiaawIcacaGLPaaadaahaaWcbeqaaiaaikdaaaaakiaawUfacaGLDbaaaSqaaiaadohacqGH9aqpcaaIXaaabaGaam4saaqdcqGHris5aaaa@46C3@

where *K *is the number of modules, *L *is the number of edges in the network, *l*_*s *_is the number of edges between nodes in modules, and *d*_5 _is the sum of the degrees of the nodes in module *s*. Since modularity is possibly affected by the different size or connectivity of the networks, it is advisable to normalize this measure with respect to the modularity of random networks with the same connectivity. These random networks are generated by swapping the connections between pairs of nodes. For instance, if the real network contains the interactions A-B and C-D, the randomized network will contain A-D and B-C. In this way, the random network maintains node degrees and connectivity.

Several algorithms have been proposed to extract modules from networks. To test the validity of our conclusions, we used two different methods to calculate modules and modularity coefficients. The algorithm of Guimerá and Nunes-Amaral [23] is based on a simulated annealing procedure, and it has been successfully used to decompose metabolic networks. Newman's algorithm [24] is based on the spectral decomposition of the eigenvectors of a modularity matrix derived from the interactions between nodes. Both methods claim to obtain optimal decomposition of the networks, and the results using both algorithms are very similar (Table 1). Guimerá's algorithm achieves slightly higher modularities, while Newman's algorithm is considerably faster, especially when dealing with big networks. The analysis of the resulting modules shows that both decompositions are similar, with 70% of the interactions belonging to the same modules. The normalized modularity coefficients are very close, regardless of the algorithm or the data source used, indicating that they are robust and not influenced by such factors.

Since we wanted to inspect the conservation of modularity when the network is reduced, the modularity of *Buchnera*'s networks was calculated either by generating a new modular decomposition for *Buchnera*, or using the same modular decomposition obtained for *E. coli *such that the modules were maintained while the nodes and interactions not present in *Buchnera *were removed. In this way, we are able to study the way in which original modules are reduced.

To check the quality and functional relevance of modules, we used data from the STRING database [26,27]. Modules with functional significance would be expected to be enriched in these interactions. Therefore, we calculated the total number of interactions per pair of proteins in STRING and, accordingly, the number of interactions per pair that would be expected within each of the modules in the network based on the size of the module. We consider that the module is validated if it is significantly enriched in STRING interactions (*p *value < 0.1).

The networks were plotted with the Cytoscape software [28]. The evaluation of functions over-represented in each of the modules (using Gene Ontology [29] 'biological process' category) was performed using the BiNGO plug-in [30]

## Additional data files

The following additional data are available with the online version of this paper. Additional data file 1 includes supplementary tables: Table S1 lists the composition of the main modules in *E. coli*, for the modular decomposition of the Butland dataset using Guimerá's algorithm; Table S2 shows the different motifs with three or four nodes found in the real networks and randomized networks; Table S3 shows the results of the modular decomposition of the Butland dataset by means of a k-means clustering algorithm, as an additional confirmation of the validity of the results; Table S4 lists the main conserved hubs in *Buchnera*, and their functions in the Butland dataset. Additional data file 2 shows the relationship between the connectivity of the nodes and their deletion in *Buchnera*'s network (Butland dataset), and the probability of the deletion of nodes as a function of the probable number of connections. Additional data file 3 illustrates three examples of hub deletion in *Buchnera*.

## Supplementary Material

Additional data file 1 Table S1 lists the composition of the main modules in *E. coli*, for the modular decomposition of the Butland dataset using Guimerá's algorithm. Table S2 shows the different motifs with three or four nodes found in the real networks and randomized networks. Table S3 shows the results of the modular decomposition of the Butland dataset by means of a k-means clustering algorithm, as an additional confirmation of the validity of the results. Table S4 lists the main conserved hubs in *Buchnera*, and their functions in the Butland dataset. Click here for file

Additional data file 2 Relationship between the connectivity of the nodes and their deletion in *Buchnera*'s network (Butland dataset), and the probability of the deletion of nodes as a function of the probable number of connections. Click here for file

Additional data file 3 Three examples of hub deletion in *Buchnera*. Click here for file
