# Supplementary material for: Modular organization in the reductive evolution of protein-protein interaction networks
Source: Genome Biol. 2007 May 28;8(5):R94. doi: 10.1186/gb-2007-8-5-r94 (PMC1929161; doi:10.1186/gb-2007-8-5-r94)
Supplement: Additional data file 1 — Table S1 lists the composition of the main modules in E. coli, for the modular decomposition of the Butland dataset using Guimerá's algorithm. Table S2 shows the different motifs with three or four nodes found in the real networks and randomized networks. Table S3 shows the results of the modular decomposition of the Butland dataset by means of a k-means clustering algorithm, as an additional confirmation of the validity of the results. Table S4 lists the main conserved hubs in Buchnera, and their functions in the Butland dataset. [file gb-2007-8-5-r94-S1.doc]

Table S1: Composition of the main modules in *E. coli,* for Butland dataset. The columns show the following information: Main functions: Functions most represented in the module; Nodes: Number of nodes in the module; Intra: Number of connections between nodes within the module; Inter: Number of connections with nodes in other modules; Perc modul: Percentage of modularity of the global network that is explained by this module; Main hub: Highly connected proteins in the module that are connected both within the module and with other modules; Stat signif: Statistical significance of the module (see methods); Deleted: Percentage of deletion, the nodes in the module that have been lost in *Buchnera* (78% for the whole network). The seven modules with the highest modularity coefficient are shown. The information for the rest of the modules that contribute less to global modularity and that are not statistically significant is shown in the last row of the table.

Table S2: Percentage of different motifs with 3 or 4 nodes, for real networks (Butland dataset) and randomized networks. The 3- and 4-node motifs are considered and counted separately.

Table S3: Additional clustering procedures have been used for modular decomposition since they have been demonstrated to be robust against the inherent noise in the experimental data. We tried to deconstruct the network into modules using a k-means clustering procedure. Since the number of clusters (modules) must be specified and this variable is unknown, we tested different numbers of clusters.

The results of analyzing the structure of the network showed that the *E. coli* network was the most modular at any level. Given the hierarchical structure of the network, the best modular decomposition was achieved by dividing the network into just five clusters or modules. However, a decomposition that renders more significant modules was achieved by deriving 19 modules, 5 of which were statistically significant (see methods).

The *Buchnera* network was always significantly less modular than that of *E. coli*. The best decomposition in terms of modularity was achieved with eight modules, five of which corresponded to the original *E. coli* modules even though only one of these was statistically significant (ribosomal proteins). The decrease in the modularity coefficient was also apparent when the network was divided into different numbers of modules, implying that the network obtained for *Buchnera* is much harder to separate into isolated components than that of *E. coli*. The constrained decomposition of the *Buchnera* network was also less modular than that of *E. coli*, and less than that observed when the *Buchnera* network was clustered independently.

Table S4: Main conserved hubs in *Buchnera* and their functions in the Butland dataset.

| Main functions | Nodes | Intra | Inter | Perc modul | Main hubs | Stat signif | Deleted |
| --- | --- | --- | --- | --- | --- | --- | --- |
| Protein biosynthesis  Ribosome | 222 | 1529 | 1304 | 33% | rluB (pseudouridine synthase B)  rplD (50S ribosomal protein L4)  deaD (RNA helicase deaD) | p<0.01 | 69% |
| DNA metabolism  Nucleotide metab. | 171 | 314 | 531 | 13% | aceE (pyruvate dehydrogenase E1)  hupA (DNA-binding protein HU-alpha)  topA (DNA topoisomerase I) | p<0.01 | 82% |
| Cell division  Response to stress  Transcription | 168 | 301 | 504 | 12% | dnaJ (Heat shock protein J)  ftsZ (Cell division protein ftsZ)  tufA (Elongation factor Tu) | p<0.1 | 81% |
| Heterogeneous | 193 | 276 | 410 | 11% | groL (groEL protein)  helD (helicase IV)  dnaK (Heat shock protein 70) | No | 81% |
| Cell cycle  Carbohydrate metabolism | 193 | 271 | 579 | 10% | trmE (probable tRNA modification GTPase)  leuS (Leucyl-tRNA synthetase)  rpe (Ribulose-phosphate 3-epimerase) | No | 80% |
| DNA polymerase  Macromolecule biosynthesis | 135 | 225 | 570 | 9% | clpA (Clp protease)  secA (preprotein translocase secA)  dnaE (DNA polymerase III alpha subunit) | No | 79% |
| Transcription  RNA polymerase | 98 | 147 | 372 | 6% | rpoA (DNA-directed RNA polymerase alpha)  nusG (Transcription antitermination protein)  nusA (Transcription elongation protein) | p<0.01 | 86% |
| Heterogeneous | 21 | 20 | 21 | 1% | lysS (lysil-tRNA synthetase) | p<0.01 | 100% |
| Heterogeneous | 12 | 11 | 13 | <1% | sucA (Alpha-ketoglutarate dehydrogenase) | p<0.1 | 58% |
| Heterogeneous | 9 | 8 | 5 | <1% | murF (D-alanyl-D-alanine ligase) | p<0.01 | 100% |

Table S1

| Motif type | Buchnera | | E. coli | |
| --- | --- | --- | --- | --- |
|  | Real | Random | Real | Random |
|  | 0.93 | 0.95 | 0.95 | 0.97 |
|  | 0.07 | 0.05 | 0.05 | 0.03 |
|  | 0.34 | 0.38 | 0.43 | 0.44 |
|  | 0.41 | 0.46 | 0.43 | 0.46 |
|  | 0.13 | 0.12 | 0.10 | 0.07 |
|  | 0.05 | 0.02 | 0.03 | 0.01 |
|  | 0.04 | 0.01 | 0.02 | <0.01 |
|  | 0.03 | 0.01 | <0.01 | <0.01 |

Table S2

| Clusters | Q_real_,  *E.coli* | Q_real_, *Buchnera* | Q_real_, *Buchnera, constrained* | Module validation, *E. coli* | Module validation, *Buchnera* |
| --- | --- | --- | --- | --- | --- |
| 3 | 0,270 | 0,101 | 0,176 | 1 | 1 |
| 5 | 0,296 | 0,100 | 0,187 | 1 | 1 |
| 8 | 0,151 | 0,123 | 0,098 | 2 | 1 |
| 10 | 0,171 | 0,113 | 0,106 | 2 | 1 |
| 12 | 0,166 | 0,092 | 0,097 | 3 | 1 |
| 15 | 0,151 | 0,093 | 0,097 | 3 | 0 |
| 19 | 0,286 | 0,086 | 0,106 | 6 | 0 |
| 25 | 0,191 | 0,108 | 0,094 | 5 | 0 |
| 30 | 0,214 | 0,082 | 0,089 | 6 | 0 |
| 100 | 0,182 | 0,055 | 0,059 | 20/70 | n/a |

Table S3

| **EC locus** | **Degree** | **Function** |
| --- | --- | --- |
| b0169 | 75 | 30S ribosomal protein S2 |
| b3319 | 74 | 50S ribosomal protein L4 |
| b3303 | 68 | 30S ribosomal protein S5 |
| b0014 | 60 | Chaperone protein dnaK |
| b3162 | 55 | ATP-dependent RNA helicase deaD |
| b2517 | 54 | protein yfgB |
| b3986 | 53 | 50S ribosomal protein L7/L12 |
| b3295 | 52 | DNA-directed RNA polymerase alpha chain |
| b3315 | 50 | 50S ribosomal protein L22 |
| b3320 | 48 | 50S ribosomal protein L3 |
| b4179 | 44 | Ribonuclease R |
| b3704 | 44 | Ribonuclease P protein component |
| b1269 | 44 | Ribosomal large subunit pseudouridine synthase B |
| b1086 | 44 | Ribosomal large subunit pseudouridine synthase C |
| b3341 | 43 | 30S ribosomal protein S7 |
| b3321 | 43 | 30S ribosomal protein S10 |
| b3231 | 43 | 50S ribosomal protein L13 |
| b3988 | 42 | DNA-directed RNA polymerase beta' chain |
| b3318 | 42 | 50S ribosomal protein L23 |
| b1718 | 39 | Translation initiation factor IF-3 |
| b3987 | 37 | DNA-directed RNA polymerase beta chain |
| b1823 | 37 | Cold shock-like protein cspC |
| b1207 | 36 | Ribose-phosphate pyrophosphokinase |
| b2606 | 34 | 50S ribosomal protein L19 |
| b0015 | 34 | Chaperone protein dnaJ |
| b0098 | 33 | Preprotein translocase secA subunit |
| b3164 | 32 | Polyribonucleotide nucleotidyltransferase |
| b0171 | 32 | Uridylate kinase |
| b3984 | 31 | 50S ribosomal protein L1 |
| b0114 | 31 | Pyruvate dehydrogenase E1 component |
| b3982 | 30 | Transcription antitermination protein nusG |
| b3317 | 30 | 50S ribosomal protein L2 |
| b3314 | 30 | 30S ribosomal protein S3 |
| b0437 | 29 | ATP-dependent Clp protease proteolytic subunit |
| b0406 | 29 | tRNA-guanine transglycosylase |
| b2960 | 28 | tRNA (guanine-N(7)-)-methyltransferase |
| b4059 | 27 | Single-stranded DNA-binding protein (SSB) |

Table S4
