# Supplementary material for: Modular organization in the reductive evolution of protein-protein interaction networks
Source: Genome Biol. 2007 May 28;8(5):R94. doi: 10.1186/gb-2007-8-5-r94 (PMC1929161; doi:10.1186/gb-2007-8-5-r94)
Supplement: Additional data file 2 — Relationship between the connectivity of the nodes and their deletion in Buchnera's network (Butland dataset), and the probability of the deletion of nodes as a function of the probable number of connections. [file gb-2007-8-5-r94-S2.ppt]

## Slide 1
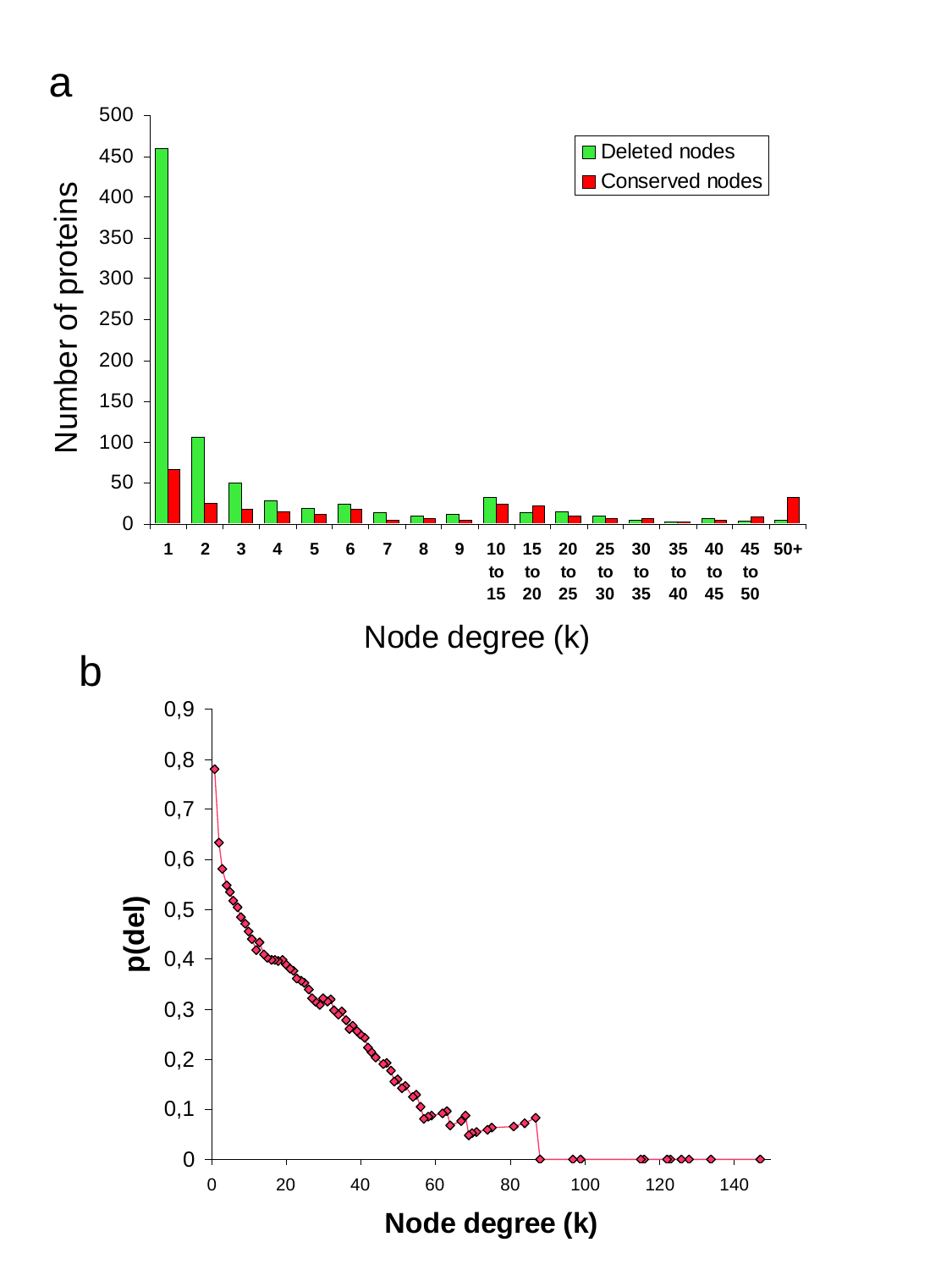

a
b

## Slide 2
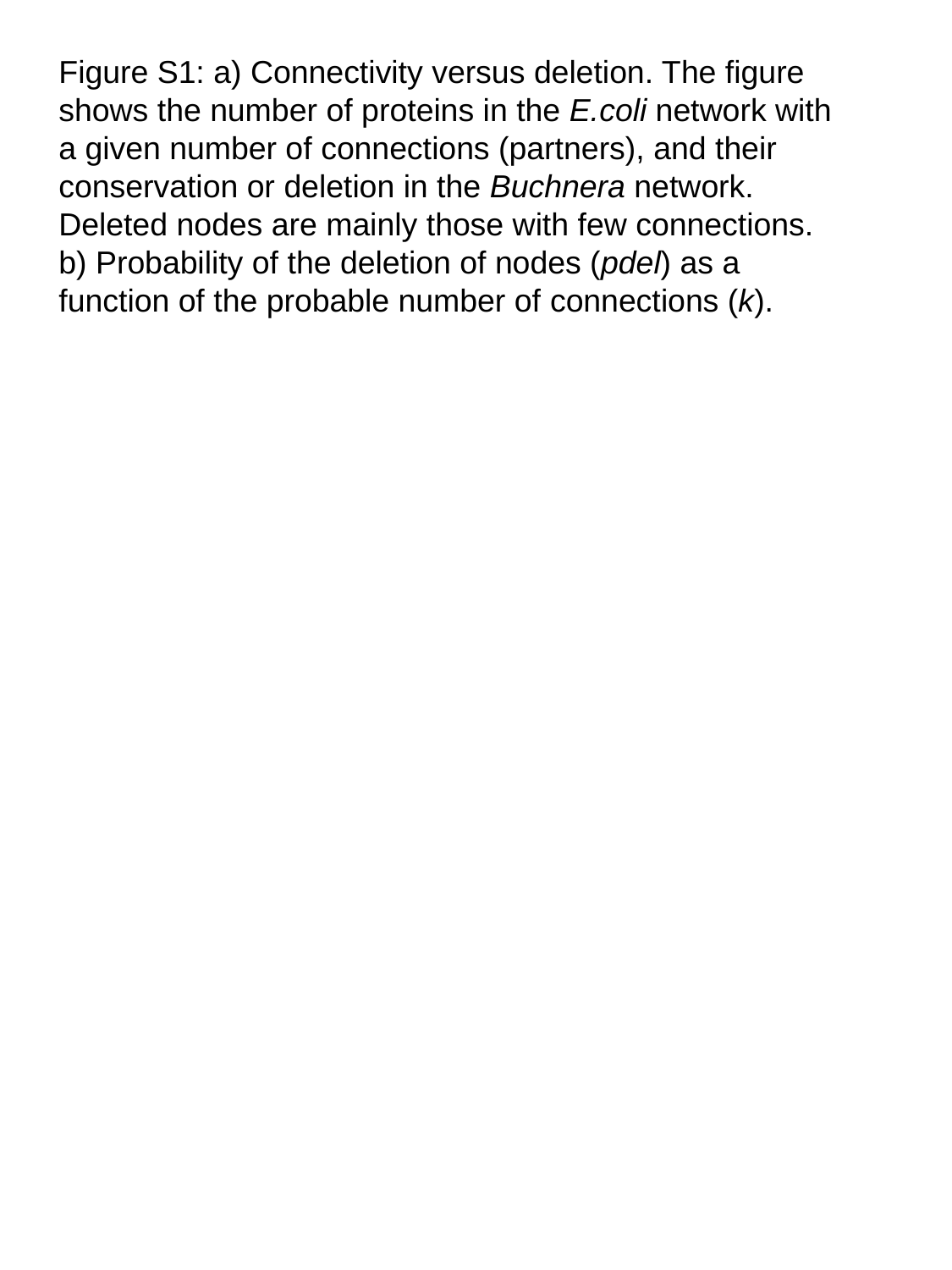

Figure S1: a) Connectivity versus deletion. The figure shows the number of proteins in the E.coli network with a given number of connections (partners), and their conservation or deletion in the Buchnera network. Deleted nodes are mainly those with few connections.
b) Probability of the deletion of nodes (pdel) as a function of the probable number of connections (k).
