# Supplementary material for: Modular organization in the reductive evolution of protein-protein interaction networks
Source: Genome Biol. 2007 May 28;8(5):R94. doi: 10.1186/gb-2007-8-5-r94 (PMC1929161; doi:10.1186/gb-2007-8-5-r94)
Supplement: Additional data file 3 — Three examples of hub deletion in Buchnera. [file gb-2007-8-5-r94-S3.ppt]

## Slide 1
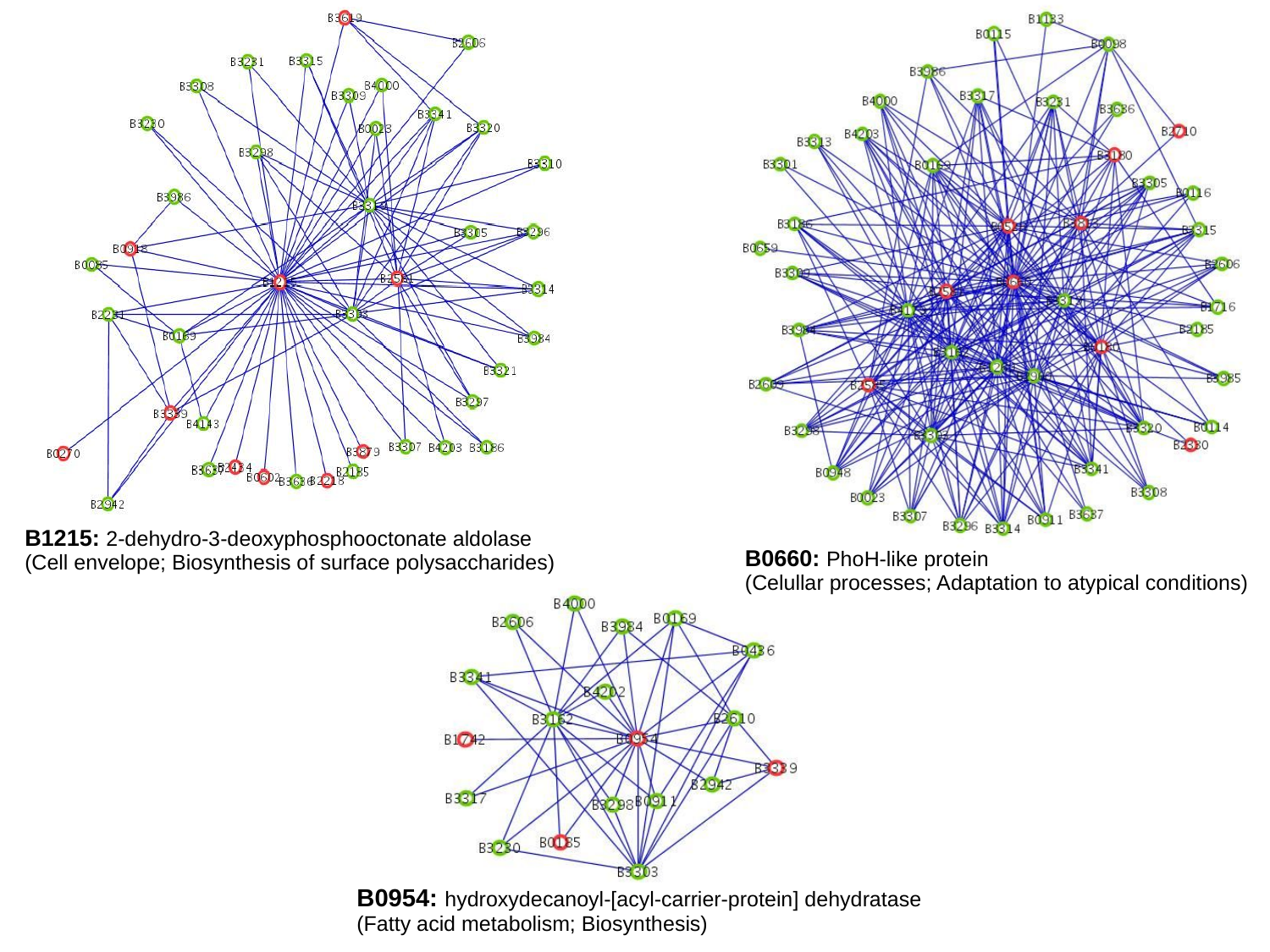

B1215: 2-dehydro-3-deoxyphosphooctonate aldolase
(Cell envelope; Biosynthesis of surface polysaccharides)
B0660: PhoH-like protein
(Celullar processes; Adaptation to atypical conditions)
B0954: hydroxydecanoyl-[acyl-carrier-protein] dehydratase
(Fatty acid metabolism; Biosynthesis)

## Slide 2
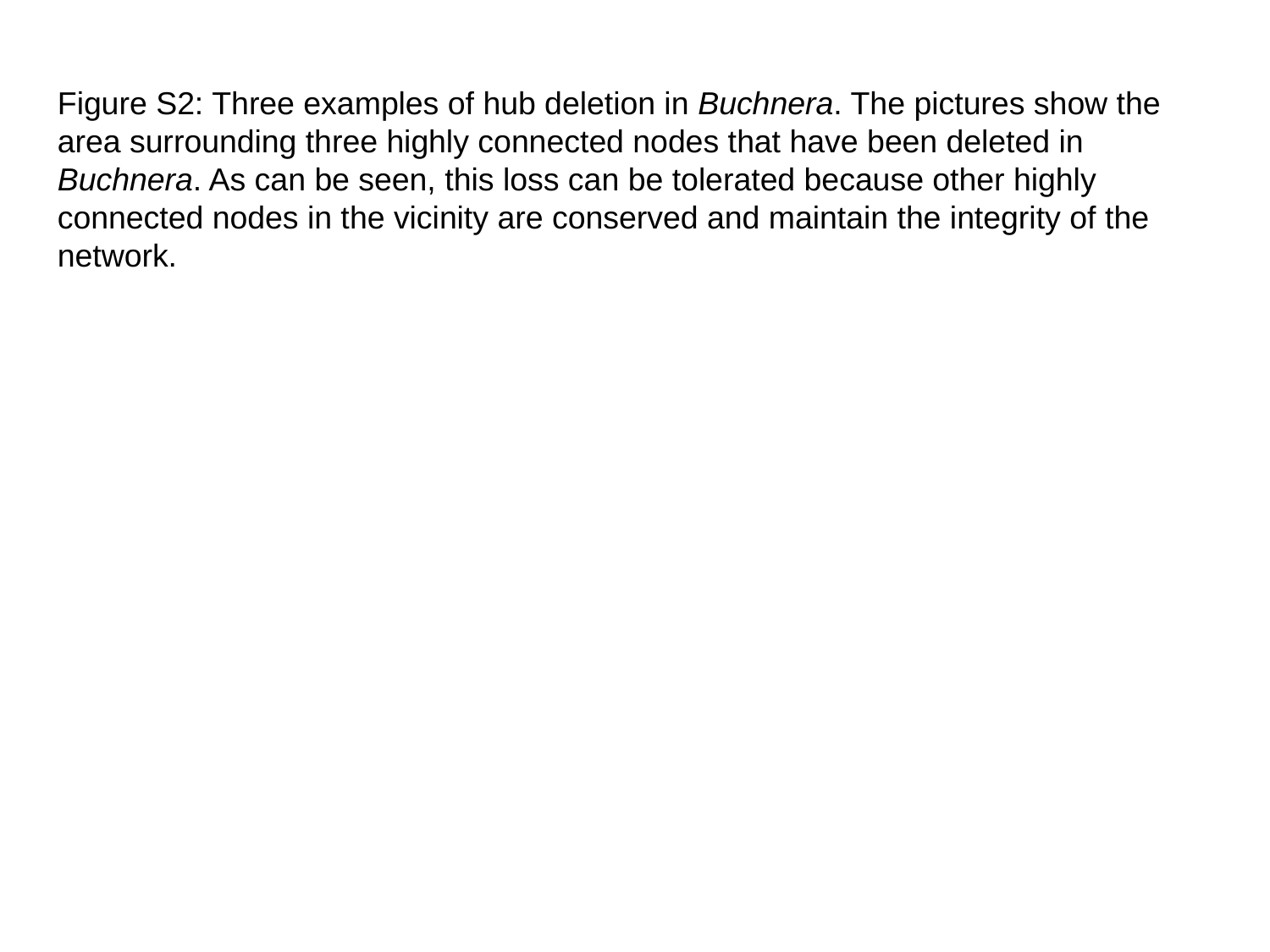

Figure S2: Three examples of hub deletion in Buchnera. The pictures show the area surrounding three highly connected nodes that have been deleted in Buchnera. As can be seen, this loss can be tolerated because other highly connected nodes in the vicinity are conserved and maintain the integrity of the network.
